# First Expert Elicitation of Knowledge on Drivers of Emergence of Bovine Besnoitiosis in Europe

**DOI:** 10.3390/pathogens11070753

**Published:** 2022-07-01

**Authors:** Claude Saegerman, Julien Evrard, Jean-Yves Houtain, Jean-Pierre Alzieu, Juana Bianchini, Serge Eugène Mpouam, Gereon Schares, Emmanuel Liénard, Philippe Jacquiet, Luca Villa, Gema Álvarez-García, Alessia Libera Gazzonis, Arcangelo Gentile, Laurent Delooz

**Affiliations:** 1 Research Unit of Epidemiology and Risk Analysis Applied to Veterinary Science (UREAR-ULiège), Fundamental and Applied Research for Animals & Health (FARAH) Center, Faculty of Veterinary Medicine, University of Liege, 4000 Liege, Belgium; juana.bianchini@uliege.be (J.B.); laurent.delooz@arsia.be (L.D.); 2 Regional Association for Animal Registration and Health (ARSIA) asbl, 5590 Ciney, Belgium; julien.evrard@arsia.be (J.E.); jeanyves.houtain@arsia.be (J.-Y.H.); 3 Laboratoire Vétérinaire Départemental de l’Ariège (LVD09), 09008 Foix, Ariège, France; veterinaire.tilleuls@hotmail.fr; 4 School of Veterinary Medicine and Science, University of Ngaoundere, Ngaoundere P.O. Box 454, Cameroon; sempouam@yahoo.fr; 5 Friedrich-Loeffler-Institut, Bundesforschungsinstitut für Tiergesundheit, Federal Research Institute for Animal Health, 17493 Greifswald, Insel Riems, Germany; gereon.schares@fli.de; 6 UMR INTHERES/DGER, Ecole Nationale Vétérinaire de Toulouse, CEDEX 03, 31076 Toulouse, France; emmanuel.lienard@envt.fr; 7 UMR INRA/DGER IHAP 1225, Ecole Nationale Vétérinaire de Toulouse, CEDEX 03, 31076 Toulouse, France; philippe.jacquiet@envt.fr; 8 Department of Veterinary Medicine and Animal Sciences, Università degli Studi di Milano, Via dell’Università 6, 26900 Lodi, Italy; luca.villa@unimi.it (L.V.); alessia.gazzonis@unimi.it (A.L.G.); 9 SALUVET Group, Animal Health Department, Faculty of Veterinary Sciences, Complutense University of Madrid, Ciudad Universitaria s/n, 28040 Madrid, Spain; gemaga@ucm.es; 10 Department of Veterinary Medical Sciences, University of Bologna, 40064 Ozzano Emilia, BO, Italy; arcangelo.gentile@unibo.it

**Keywords:** bovine besnoitiosis, *Besnoitia besnoiti*, drivers, expert elicitation, cattle, multi-criteria decision analysis (MCDA), clustering analysis, sensitivity analysis

## Abstract

Bovine besnoitiosis (BB) is a chronic and debilitating parasitic disease in cattle caused by the protozoan parasite *Besnoitia besnoiti*. South European countries are affected and have reported clinical cases of BB. However, BB is considered as emerging in other countries/regions of central, eastern and northern Europe. Yet, data on drivers of emergence of BB in Europe are scarce. In this study, fifty possible drivers of emergence of BB in cattle were identified. A scoring system was developed per driver. Then, the scoring was elicited from eleven recognized European experts to: (i) allocate a score to each driver, (ii) weight the score of drivers within each domain and (iii) weight the different domains among themselves. An overall weighted score was calculated per driver, and drivers were ranked in decreasing order of importance. Regression tree analysis was used to group drivers with comparable likelihoods to play a role in the emergence of BB in cattle in Europe. Finally, robustness testing of expert elicitation was performed for the seven drivers having the highest probability to play a key role in the emergence of BB: i.e., (i) legal/illegal movements of live animals from neighbouring/European Union member states or (ii) from third countries, (iii) risk of showing no clinical sign and silent spread during infection and post infection, (iv) as a consequence, difficulty to detect the emergence, (v) existence of vectors and their potential spread, (vi) European geographical proximity of the pathogen/disease to the country, and (vii) animal density of farms. Provided the limited scientific knowledge on the topic, expert elicitation of knowledge, multi-criteria decision analysis, cluster and sensitivity analyses are very important to prioritize future studies, e.g., the need for quantitative import risk assessment and estimation of the burden of BB to evidence and influence policymaking towards changing (or not) its status as a reportable disease, with prevention and control activities targeting, firstly, the top seven drivers. The present methodology could be applied to other emerging animal diseases.

## 1. Introduction

Bovine besnoitiosis (BB) is a chronic and a debilitating parasitic disease of cattle caused by a protozoan parasite called *Besnoitia besnoiti* [1]. *Besnoitia* spp. belong to the phylum Alveolata, subphylum Apicomplexa, a diverse group of largely parasitic protozoa of considerable veterinary and medical importance. Members include the genera *Plasmodium*, *Cryptosporidium*, *Eimeria*, *Isospora*, *Sarcocystis*, *Toxoplasma*, *Neospora*, *Theileria* and *Babesia* [2]. There are several recognized species (*B. besnoiti*, *B. caprae*, *B. bennetti* and *B. tarandi*) in the genus *Besnoitia* [3] infecting domestic and wild ungulates (cattle, goats, equids and cervids, respectively). This study focuses exclusively on drivers of emergence of BB due to *B. besnoiti* in Europe. Cattle are the predominant intermediate hosts of *B. besnoiti* in Europe and it is also reported to be able to infect antelopes [4] and roe deer (*Capreolus capreolus*) [5]. The life cycle of the parasite species infecting ruminants is not completely known, especially with regard to the definitive host [6]. In addition, a recent survey reported a *B. besnoiti*-like sequence (99.57% homology) from 4 out of 187 red foxes (*Vulpes vulpes*) feces tested in Spain, necessitating further investigation to confirm or refute the role of this species in the epidemiology of *B. besnoiti* in Europe [7].

There is evidence that biting insects or the re-use of a contaminated needle for group injections can mechanically transmit *B. besnoiti* [8,9,10]. In Europe, different species of blood-sucking insects, such as tabanid species and stable flies (*Stomoxys calcitrans*), may transmit *B. besnoiti* mechanically from chronically or asymptomatic infected cattle. The vector insects remain infectious for only a few hours after their blood meal on a carrier animal [11,12]. The time period during which vectors remain infectious after blood meal on an infected animal is short and varies according to species. It is 1 h in the case of *S. calcitrans*, 3 h for *Glossina brevipalpis* and 24 h for tabanids [11]. Although transmission by mosquitoes (*Culex simpsoni* and other unidentified *Culex* spp.) has not been demonstrated, it has been shown that mosquitoes are able to ingest *B. besnoiti* from bovine skin and that the parasites remain infectious for hours (50 h). Thus, their role as vectors remains not well established yet [11]. Another study showed that *Stomoxys* were able to transmit in vitro parasites 48 h after their last meal on infected cattle [12].

This disease presents two distinct phases: a first acute phase followed by a chronic phase. In the acute phase, clinical signs are non-specific and do not really help in the diagnosis. Otherwise, in the chronic phase, clinical signs could be very suggestive of BB [13], but this is only true for a small proportion of animals. Indeed, a large proportion of concerned animals are sub-clinically infected without clinical signs. Those animals represent a huge risk for parasite transmission on farm [14,15,16]. Relying only on clinical signs for detection can lead to misdiagnosis, as BB can be confused with other infectious diseases characterized by similar clinical signs [13]. It is therefore essential to carry out confirmatory laboratory tests.

Different laboratory diagnostic methods (histopathology, polymerase chain reaction (PCR), immunofluorescence antibody test (IFAT), Western blot (WB) and enzyme-linked immunosorbent assay (ELISA)) are available for detection of *B. besnoiti* infection in cattle. The use of one of these methods or their combination depends on the animal’s and corresponding herd’s clinical status [14]. 

Concerning BB control, there is no effective drug or vaccine available, although in South Africa and Israel, a live-attenuated vaccine has been used [17]. Detection followed by culling is also a management option. However, the lack of detection/regulation in animals’ movements within a country and between countries has led to its rapid spread from Western Europe towards eastern countries and to the north [14,17]. There are few references available concerning control measures against insects (vectors) related specifically to BB. In 1968, Bigalke demonstrated the possibility of vector control [11]. Some pyrethroids are active on stomoxes, but controlling the latter becomes difficult because of insecticide resistance development [18,19]. For tabanids, only a short time effect of pyrethroid insecticides has been cited [20] and they are considered far less effective than similar applications targeting other vectors (e.g., mosquitoes).

Outbreaks of BB have been reported in Africa, mainly in the South [14]. BB has also been reported in Israel, Kazakhstan, the People’s Republic of China, India and Venezuela [21,22]. In Europe, the disease is endemic (referring to a clinically expressed or non-expressed disease that occurs regularly in animals in a given area) in Spain, Portugal, Italy and France [21,23,24]. In endemic areas, there are very few studies on the economic impact of BB. However, recently, a paper has revealed an association with a higher milk somatic cell count and BB, which can induce important economic losses [25].

Several outbreaks have been reported in European non-endemic countries [22] such as Germany [26], Switzerland [27], Croatia [28], Hungary [29], Ireland [30] and Belgium [31,32]. A range of reasons could explain this new scenario, including the animal trade, management practices such as sharing pastures, and climate change by modification of the vector activity [33]. Indeed, disease emergence is related to the joint presence of several factors, called “drivers”. The knowledge of these drivers is crucial to properly understand host–pathogen–environment interactions [34].

The aim of this study was to investigate, for the first time, the drivers of emergence of BB in Europe using expert elicitation. Multi-criteria decision analysis (MCDA) was chosen to allow systematic integration of information from a range of sources [35] and improve repeatability and transparency [36].

## 2. Results

### 2.1. Response Rate and Field of Expertise Mobilised by the Experts

Eleven European professionals with recognized scientific knowledge and/or field knowledge or experience on BB in cattle were contacted and all agreed to participate. The fields of expertise were summarized in Appendix Table A1.

### 2.2. Estimating the Overall Weighted Score and Ranking of Drivers of Bovine Besnoitiosis in Cattle 

**Figure 1:**
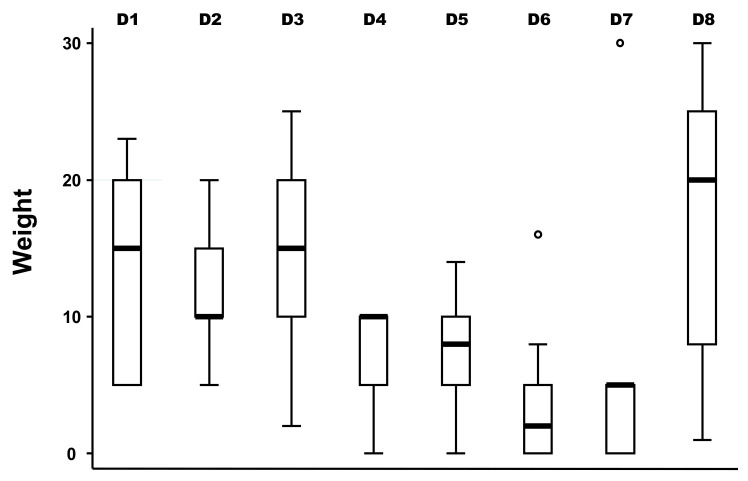
Boxplot of the relative importance of the eight domains of bovine besnoitiosis in cattle (N = 11 experts). Legend: The solid bold line represents the median of the score distribution between the different experts; the solid lines below and above each rectangle represent, respectively, the first and the third quartiles; adjacent lines to the whiskers represent the limits of the 95% confidence interval; small circles represent outside values. The eight domains of drivers are: D1, pathogen/disease characteristics; D2, distance of outbreaks (spatial-temporal scales); D3, ability to monitor, treat and control the disease; D4, European farm characteristics; D5, changes in climate conditions; D6, wildlife interface; D7, human activity; and D8, economic and trade activities.

The medians of the weight between domains of drivers as well as for the different drivers were not equal according to the non-parametric Kruskal–Wallis test (Chi-squared test = 30.1 with 7 d.f. and α = 0.05, *p*-value = 0.0001; and Chi-squared test = 119.1 with 49 d.f. and α = 0.05, *p*-value = 0.0001, for the weights between domains and weights of the different drivers, respectively) (Figure 1). 

The median of the weight of the domain D6 (wildlife interface) was significantly lower than the median of the other domains (bootstrapped regression; *p*-value < 0.001). 

**Figure 2:**
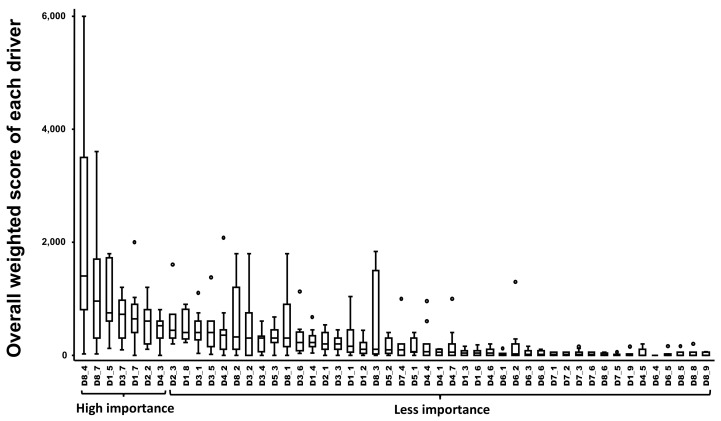
Ranking of the median overall weighted score for each potential driver of bovine besnoitiosis in cattle. (Boxplot based on 11 experts). Legend: the X-Axis represents the drivers with the following codification: D1 to D8 refer to the eight domains of drivers and D1_1 to D8_9 refer to a specific driver (for the codification, see Appendix B), small circles represent outside values. The relation to Figure 3 was provided by the group named as having, respectively, “more importance” and “less importance” in bovine besnoitiosis emergence.

Ten drivers out of 50 were ranked as having the highest probability to play a key role in the emergence of BB in Europe. Indeed, the following drivers were ranked in a descending order of importance: the most likely influence of (il)legal movements of live animals (i.e., cattle) from neighboring/European Union member states (MS) (D8–4) or Third countries (a country that is not a member of the European Union as well as a country or territory whose citizens do not enjoy the European Union right to free movement) (D8–7) for the disease to (re)emerge in a given country, the risk of showing no clinical sign and silent spread during infection and post infection (D1–5) and as consequence, the difficulty of detecting the emergence (D3–7), the existence of vectors and its potential spread (D1–7), the European geographic proximity of the pathogen/disease to the country (D2–2), the animal density of farms with extensive (small holders with a few animals) versus intensive farming (D4–3), the disease’s last reported case in Europe (D2–3), the mode of transmission of the pathogen (D1–8) and the problem of the ability of preventive/control measures to stop the disease from entering the country or spreading, excluding treatment, vaccination and vector(s)/reservoir(s) control (D3–1) (Figure 2).

### 2.3. Cluster Analysis 

**Figure 3:**
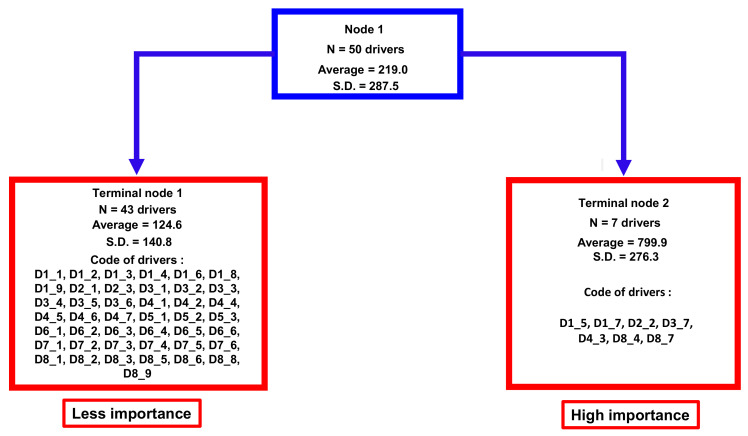
Aggregation of drivers of bovine besnoitiosis in cattle using the score, into two homogenous groups using a regression tree analysis. Legend: N, number; Average, average score; SD, standard deviation; D1 to D8 refer to the eight domains of drivers and D1_1 to D8_9 refer to a specific driver (for the codification, see Appendix B).

Two significantly different clusters were identified by regression tree analysis (Figure 3) (non-parametric Kruskal–Wallis equality-of-populations rank test; Chi-squared test = 102 with 1 d.f. and α = 0.05; *p*-value = 0.0001). These two clusters were classified as having “less importance” with 43 drivers and “more importance” with 7 drivers (D1_5, D1_7, D2_2, D3_7, D4_3, D8_4, D8_7), respectively.

### 2.4. Sensitivity Analysis of the Impact of Experts on the Final Ranking of Bovine Besnoitiosis Top Drivers of Emergence in Cattle

The result of the sensitivity analysis indicated that irrespective of the expert ignored, ignoring an expert only had no effect on the ranking of top 5 or 7 drivers (i.e., drivers included in the cluster with significantly more importance) considering a change of one or two ranks, respectively. These results were confirmed using a Kruskal–Wallis equality-of-populations rank test on experts for the top five or seven drivers identified. Indeed, results were very conclusive and respectively: chi-squared = 9.61 with 10 d.f. and probability = 0.48 (top five drivers) and chi-squared = 11.17 with 10 d.f. and probability = 0.34 (top seven drivers).

## 3. Discussion

Fifty drivers of BB in cattle were ranked and aggregated into two homogenous groups according to the present expert elicitation. Only the first ten most important ranked drivers will be further discussed with a focus on the seven categorized in the “more importance” node. In addition, for ranking of the first seven drivers, there was no expert effect when assessed by sensitivity analysis, indicating an acceptable robustness of the elicitation for the seven drivers included in the first node.

The first and second most important drivers were the influence of (il)legal movements of live animals from neighboring/European Union MS (D8–4) or from Third countries (D8–7), respectively. In European countries there are currently fewer movements of live animals originating from Third countries (note that BB was also reported in Africa and Asia) than from neighboring/European Union MS, explaining the difference in rank of these two drivers (see also international trade statistics, available at the following URL address: https://www.trademap.org/tradestat/Index.aspx; accessed on 15 December 2021). However, the animal trade from Africa to Europe may explain why the disease appeared in Europe by the end of 19th century and the beginning of 20th century in Portugal and France. In addition, there are very few studies available on the estimation of illegal movements of live animals in the scientific literature (e.g., [37,38]). Nevertheless, a proper estimation of the relative importance of illegal movements of live animals and their introduction pathways is deemed essential to set up risk-based awareness, prevention and surveillance programs that correspond to reality [34]. Direct (isolation of the protozoan parasite and real-time polymerase chain reaction (rtPCR)) and indirect (IFAT, WB and ELISA) diagnostic tests have been set up for BB [14]. Some commercial assays permit to implement a proper testing strategy in order to control the trade of live animals and to certify the sanitary BB status of the herd of origin. In order to identify mitigation measures, we strongly recommend developing a quantitative import risk assessment (QIRA) modelling similar to those developed for Lumpy skin disease that involved live bovines as well as *S. calcitrans* as a mechanic vector [39,40]. These previous studies can serve as a basis for further modelling development. 

The third and the fourth most important drivers were related to the risk of showing no clinical sign and silent spread during infection and post infection (D1–5) and as consequence, the difficulty to detect the emergence (D3–7). The disease is expressed only in the most susceptible animals [9,41,42]. In the chronic phase, cutaneous lesions and patognomnic scleral cysts may be helpful for diagnosis and also surveillance. However, many animals are sub-clinically infected with low parasite loads and they may act as parasite carriers which can only be diagnosed by serological tools [43]. In endemic areas, clinical cases are observed between 1–10% of the new infections but between 15–20% in the case of *B. besnoiti* infections in areas where the disease is emerging [44]. Moreover, during the first weeks following infection, acutely infected animals may be difficult to be clinically diagnosed due to non-specific signs [21]. In addition, several other diseases should be considered in the differential diagnosis according to the stage of the BB such as malignant catarrhal fever, bovine granulocytic ehrlichiosis, bluetongue, bovine respiratory disease, photosensitization, scabies or zinc deficiency [13]. Indeed, clinical surveillance of BB is not fully efficient, and it is essential to carry out confirmatory laboratory tests [21]. There is no formal gold standard test for BB but four tests are frequently used to confirm a clinical suspicion of BB: rtPCR and serological tests (IFAT, ELISA and WB). For WB, the sensitivity (Se) and specificity (Sp) are close to 100%. The Se and Sp of the IFAT are close to 100% and 95%, respectively. Depending on the ELISA used, their Se and Sp are generally > 97% and >93%, respectively [45]. The Se and Sp of the rtPCR are around 90% and >99% [46]. A previous study recommended also a mandatory active surveillance system via a systematic analysis of all imported animals originating from areas at risk [32]. Research is recommended to develop more commercial accurate laboratory assays and decision-making trees able to help the diagnostic of BB.

The fifth most important driver was the existence of vectors and their potential spread (D1–7). The knowledge of different species of blood-sucking insects in a country, their distribution and frequency over time, and the time-period during which vectors remain infectious after a blood meal on an infected animal [10,11,12] are of prime importance to develop QIRA modelling [39,40]. In addition, due to the presence of mechanical vectors of BB (i.e., *S. calcitrans* and tabanids) in Europe, the seasonality of BB was previously reported as playing a major role in disease epidemiology [18]. The inclusion of seasonality should be valuable for further development of a QIRA modelling.

The sixth most important driver was geographic proximity between a specific non-endemic country and a specific endemic country of origin (D2–2). This driver is related to the third and the fourth ones because if a disease is notifiable, it is easier to secure the trade. Threat analysis and QIRA modelling should be appropriate responses to deal with this driver [39,40].

The seventh most important driver is related to the animal density of farms with extensive (small holders with a few animals) versus intensive farming (D4–3). Density of farms is a driver of spread of a disease, especially if mechanical vectors are present and if these vectors are able to transmit parasites for few hours after their last (interrupted) blood meal on infected cattle [12].

The eighth most important driver is the last reported case of the disease in Europe (D2–3). This driver can be related to the fact that BB is currently not a reportable disease in most of the affected countries. Several criteria to include a disease as reportable exist among which the most important is its zoonotic character (that was not the case for BB according to [17]) and its significant health impacts, taking into account the occurrence and severity of the clinical signs, including direct production losses and mortality [47]. Despite several papers reporting economic concerns related to BB (e.g., [28,32]), factual data on the burden of the disease and its translation to monetary losses are completely lacking [17]. We strongly recommend estimating the economic burden of BB in order to convince policy makers to take action (or not) whether to include BB as a notifiable disease based on factual data.

The ninth most important driver was related to the mode of transmission of the pathogen (D1–8). There is evidence that several biting insects can mechanically transmit *B. besnoiti* [8,9,10,11] but the entire life cycle remains unknown and especially the definitive host [6]. The intra-herd transmission of BB is generally intense but weak between herds [48]. However, no information of the basic reproductive number for BB is known. Currently, in Europe, there is no strong evidence of the role of the wildlife in BB [49,50]. More studies are needed.

The tenth most important driver was related to the problem of the ability of preventive/control measures to stop the disease from entering the country or spreading (D2–1). Recent studies recommended the awareness of decision-makers about the need for an appropriate prevention and control policy, law enforcement and the implementation of necessary measures to avoid BB becoming endemic in non-endemic countries [32,51,52,53]. As biosecurity measures, a quarantine and a systematic screening of all imported animals originating from areas at risk can be proposed [32]. In addition, in South Africa and Israel, live-attenuated vaccines were used [17]. Other valuable preventive/control measures should be identified using networking permitting sharing of information and experiences between researchers/veterinarians and literature search, especially systematic review and meta-analyses and using an evidenced-based approach. 

As an example of a recent advance, real-time PCR on skin biopsies permitted the detection of super-spreaders in BB [1] and identification/elimination of these super-spreaders contribute to disease control in heavily infected herds. The control of stable flies can be difficult by the development of insecticide resistance [19] and nothing is known about the eventual resistance of horseflies to insecticides (or even their effectiveness). In addition, the lack of repellents with long lasting activity in livestock hampers ecto-parasite control. Moreover, regular treatments are not feasible in extensive husbandry systems.

Considering the European spread in time and space of the BB, the importance of live-animal trade between European countries (endemic versus non-endemic), the fact that notification of the disease is currently not mandatory, the large proportion of sub-clinically infected animals (but at risk), the need for affordable confirmatory tests, and the climatic changes that affect and alter the habitats and population dynamics of vectors, the BB is becoming a concern and needs more collective efforts to limit its spread and its impacts.

## 4. Materials and Methods

The methodology followed in this expert elicitation of knowledge is the same as previously published [33,34] for other emerging diseases but is adapted for BB. For transparency, the method is detailed below.

### 4.1. Species Included

The objective was to prioritize the drivers of BB in Europe. Using the following algorithms on 12 December 2021 (((bovine besnoitiosis [Title/Abstract]) OR (*Besnoitia besnoiti* [Title/Abstract]) OR (*B. besnoiti* [Title/Abstract])) AND (cattle [Title/Abstract]) AND (Europe [Title/Abstract])), search strings were conducted in PubMed (US National Library of Medicine, National Institutes of Health). The results of the search (N = 47 articles from 2009 through 2021) showed that three review paper were produced; other papers were related to field/epidemiological surveys (N = 20), biology studies (N = 10), diagnosis (N = 8), experimental studies (N = 3), treatments (N = 2), and vectors (N = 1).

### 4.2. Questionnaire Design

To determine the main drivers of BB emergence, a questionnaire was used. A driver was defined as a factor that has the potential to directly or indirectly precipitate (“drive”) or lead to the emergence of BB in cattle. A former questionnaire made to rank (re-)emergence of animal diseases based on drivers [33] was adjusted for bovine besnoitiosis in cattle. Fifty drivers were established and classified in eight different domains (Appendix B). The domains (D) were: (D1) disease/pathogen characteristics (N = 9 drivers); (D2) distance of Europe (spatial-temporal scales) (N = 3 drivers); (D3) ability to monitor, treat and control the disease (N = 7 drivers); (D4) Farm/European characteristics (N = 7 drivers); (D5) changes in climatic conditions (N = 3 drivers); (D6) wildlife interface (N = 6 drivers); (D7) human activities (N = 6 drivers); and (D8) economic and trade activities (N = 9 drivers).

These were formatted in an Excel^®^ (Microsoft, Redmond, WA, USA, 2016) file with one spreadsheet per domain, each domain harbouring its respective drivers. Each driver had a score with its definition, which could range from 0 to 4 or 1 to 4 and an intra driver weight point. A last spreadsheet was added, in which the 8 domains were listed, with an inter-domain weight. 

### 4.3. Expert Elicitation on Drivers Used to Assess the Emergence of Bovine Besnoitiosis in Europe 

An expert elicitation of knowledge was conducted, which consisted of gathering the opinion of people with recognized scientific expertise and/or experience in the field of BB in cattle (Appendix A). For guidance purposes, an explanatory letter accompanied the questionnaire that each expert had to fill out (Appendix C). Each expert was contacted personally and responded individually to the questionnaire. Data generated by the elicitation were based on the individual values provided by experts in order to capture the degree of variability of experts’ knowledge. The elicitation was performed in one month.

### 4.4. Scoring and Weighting System

The elicited experts were asked to provide three types of information. First, they were asked to score the drivers (as established in Appendix B). For each driver, the higher the score, the higher the driver’s chance to contribute to the emergence of BB in cattle. Uncertainty score was not asked due to lack of evidence-based data on BB in cattle at this stage. Secondly, experts were requested to weight each driver within a specific domain (intra-domain weight). This relative weight was determined using the Las Vegas technique [54]. Briefly, experts were given a number of points to be distributed between the drivers according to their importance in the specific domain. If all the drivers of a given domain had been considered as equivalent by experts, each of them would have received the same score. Lastly, the relative importance of each domain was subsequently weighted by experts (inter-domain weight).

### 4.5. Calculation of an Overall Weighted Score for Each Driver and Ranking Process

To obtain the overall score per driver, an aggregation method that combined the two types of weighting (i.e., the intra- and inter-domain) was used. First, the driver score (coefficients attributed by experts) was standardized by dividing it by the number of possibilities. Indeed, some drivers were allocated coefficients from 0 to 4 (5 possibilities) and others from 1 to 4 (4 possibilities). Afterwards, this standardized score was multiplied by the intra-domain weight and the inter-domain weight, as given by the expert. These results led to an overall weighted score for each driver and per expert:

OWSDri = SDri × WDri × WDoj(1)

In this formula, OWSDri = overall weighted score for a specific driver; SDri = standardized score for a specific driver; WDri = intra-domain weight for a specific driver; WDoj = inter-domain weight for a specific driver included in a specific domain. Furthermore, all drivers were ranked based on the median overall weighted score obtained for each driver and taking into account the answers of all the experts who answered the questionnaire. The statistical difference of the median, depending on the specific driver or the group of drivers considered, was assessed through a non-parametric Kruskal–Wallis equality-of-populations rank test (State SE 14.2; StataCorp, College Station, TX, USA).

### 4.6. Cluster Analysis

A cluster analysis was carried out using a regression tree analysis (Salford Predictive Modeler^®^, Version 8.2, Salford Systems, San Diego, CA, USA). The median overall weighted score (median OWSDri) being a continuous variable, the aim was to obtain groups of drivers with minimal within-group variance, with comparable likelihood to play a role in the emergence of BB in cattle. In addition, the statistical difference between medians after grouping drivers in clusters was assessed using a non-parametric Kruskal–Wallis equality-of-populations rank test. Indeed, each driver was characterized by a median (based on all experts’ answers), then drivers were grouped. The test allowed highlighting of potential significant differences between groups, in terms of driver medians, after clustering.

### 4.7. Sensitivity Analysis to Test the Robustness of the Expert Elicitation

In order to identify whether the ranking of BB drivers of emergence was influenced by the choice of experts, a sensitivity analysis was performed on the top five and top seven drivers. First, we started by ranking the drivers using the obtained median OWSDri. Second, an expert was excluded from the analysis and the ranking of the drivers was carried out using the same methodology as previously described. This was done expert by expert. Third, we counted the number of changes in the ranking, for each driver, only considering changes equal or more than one rank. These results were confirmed using a Kruskal–Wallis equality-of-populations rank test on experts.

## 5. Conclusions

Since scientific knowledge on drivers of emergence of BB in cattle is still incomplete and associated uncertainty is high, expert elicitation of knowledge and multi-criteria decision analysis, in addition with clustering and sensitivity analyses, allowed the identification of seven drivers of more importance on which to focus on future studies. The transport of live cattle asymptomatic carriers seems to be a key factor of introduction and spread of BB. Indeed, further quantitative import risk assessment and estimation of economic burden of BB are highly recommended. This expert elicitation of knowledge should be also refined in the coming years when more evidence data will be available. In this case, addition of an uncertainty index should be recommended during elicitation. The present methodology could be applied to other emerging animal diseases. The application of this methodology to a specific disease also allows highlighting or not the need for more investigations.

## Data Availability

The data that support the findings of this study are available from the corresponding author upon request.

## Appendix A. Profile of Experts Involved in the Elicitation of Knowledge (N = 11)

**Table A1:** Profile of experts involved in the elicitation of knowledge (N = 11).

Last Name	First Name	Gender	Institution	Country	Field of Expertise
Álvarez-García	Gema	Female	Complutense University of Madrid	Spain	Animal health: Parasitology and parasitic diseases
Alzieu	Jean-Pierre	Male	Veterinary Laboratory of the department of Ariège	France	Animal parasitology
Delooz	Laurent	Male	Regional Association for Animal Registration and Health	Belgium	Animal disease epidemiology
Evrard	Julien	Male	Regional Association for Animal Registration and Health	Belgium	Animal disease project management
Gentile	Arcangelo	Male	Department of Veterinary Medical Sciences, University of Bologna	Italy	Bovine internal medicine
Houtain	Jean-Yves	Male	ARSIA	Belgium	Animal disease management
Jacquiet	Philippe	Male	Ecole Nationale Vétérinaire de Toulouse	France	Parasitology, parasitic diseases, applied zoology and tropical parasitology
Gazzonis	Alessia Libera	Female	Università degli Studi di Milano	Italy	Veterinary parasitology, parasitic diseases
Liénard	Emmanuel	Male	Ecole Nationale Vétérinaire de Toulouse	France	Parasitology, parasitic diseases and applied zoology
Schares	Gedeon	Male	Friedrich-Loeffler-Institut	Germany	Animal health
Villa	Luca	Male	Università degli Studi di Milano	Italy	Veterinary parasitology, parasitic diseases

## Appendix B. Domains with Each Defined Driver and Their Respective Defined Scores

**Table A2:** Domains with each defined driver and their respective defined scores (Adapted form [24]).

Domain D1. Disease/Pathogen Characteristics		
D1_1	Current Knowledge of the Pathogen.	
	**Score 0**	
	**Score 1**	**Very high:** deep scientific knowledge on the pathogen, extensive scientific literature available on its biology (transmission mode, knowledge on vector(s), infectivity, etc.)
	**Score 2**	**High:** detailed scientific knowledge on the pathogen but conflicting scientific results; some elements of the pathogen’s biology are still not elucidated
	**Score 3**	**Moderate:** limited scientific knowledge on the pathogen agent because it is still under characterization; pathogen recently discovered/isolated but belonging to a well-known and studied family of pathogens; the pathogen is characterized by multiple variants not characterized yet
	**Score 4**	**Low:** lack of scientific knowledge on the pathogen (multiplication, infectivity, incubation period, transmission mode, etc.); pathogen agent recently discovered and emerging
**D1_2**	**The current species specificity of the causing agent of the disease**	
	**Score 0**	
	**Score 1**	**Low:** Only one host is involved belonging to the same family. e.g., only cattle, small ruminants, swine
	**Score 2**	**Medium:** two species involved
	**Score 3**	**High:** three species involved
	**Score 4**	**Very high:** affects more than 3 types of families
**D1_3**	**Genetic variability of the infectious agent**	
	**Score 0**	**Negligible:** The infectious agent is genetically stable
	**Score 1**	**Low:** The genetic variability is low therefore it has a low effect in the (re)emergence of the pathogen
	**Score 2**	**Medium:** The pathogen can be considered with a medium genetic variability
	**Score 3**	**High:** The pathogen is considered with a high genetic variability
	**Score 4**	**Very high:** Very high genetic instability (e.g., high mutation rate, re-assortment and recombination). Potentially the three phenomena can characterise the pathogen’s evolution
**D1_4**	**Transmission of the agent in relation of the possible spread of the epidemic or pandemic (i.e. ease/speed of spread)**	
	**Score 0**	
	**Score 1**	**Low:** Low and slow transmission within groups of animals. Between a group of animals only if an infected animal is introduced, close contact
	**Score 2**	**Medium:** Medium ease/speed transmission within the group of animals and between groups of animals
	**Score 3**	**High:** Fast transmission within a group of animals. In a short period of time all animals of the group are infected. Adjacent groups become infected fast
	**Score 4**	**Very High:** Very fast and high transmission within the groups of animals and between groups of animals. A complete area is infected in a very short period of time
**D1_5**	**Risk of showing no clinical signs and silent spread during infection and post infection**	
	**Score 0**	**Null:** Silent spread is not part of the pathogen’s characteristics
	**Score 1**	**Low:** Very short incubation period and signs of infections easily detected/recognised.
	**Score 2**	**Moderate:** Very short incubation period and signs of infection are NOT easily detected/recognised
	**Score 3**	**Medium:** Long incubation period, clinical signs are not characteristics and therefore specific diagnosis is necessary to detect infection.
	**Score 4**	**Very high:** Long incubation period. Disease/infection shows not clinical symptoms during the infectious period. Chronic shedder
**D1_6**	**Wildlife reservoir and potential spread from it**	
	**Score 0**	**Null:** no known wildlife reservoir. Disease has never been reported in wildlife species
	**Score 1**	**Low:** few clinical cases have been reported in wildlife and no transmission to livestock has ever been documented
	**Score 2**	**Moderate:** wildlife is a reservoir of the disease but only accidental spillovers to livestock have been reported
	**Score 3**	**High:** wildlife is a reservoir of the pathogen/disease but certain environmental conditions (e.g. floods, farms crossing the farmland-bush division, etc) have to occur for the pathogen/disease to (re)emerge in livestock
	**Score 4**	**Very high:** Disease establishes itself in wildlife as a reservoir and very hard to eradicate it from wildlife. Livestock easily gets infected with the contact with wildlife
**D1_7**	**Existence of vectors (vertebrate and invertebrate, e.g., mosquitoes, bats, rodents, ticks, midges, culicoides) and potential spread**	
	**Score 0**	**Null:** No known vector
	**Score 1**	**Low:** only one type of vector is present in the country but it’s role in the transmission is presumed low (has not been assessed to date)
	**Score 2**	**Moderate:** only one type of vector exists in the country and has only been suspected as source and spread of disease
	**Score 3**	**High:** only one competent vector is present and can carry and spread the disease
	**Score 4**	**Very high:** more than one type of vector can carry and spread the disease and are found spread in most of the territory
**D1_8**	**Transmission of the pathogen**	
	**Score 0**	
	**Score 1**	**Low:** Animals only are infected by direct close contact with other infected animals and vertical transmission
	**Score 2**	**Moderate:** transmission by direct and indirect contact only (e.g., through vehicles, clothes, instruments) or non-flying vector (e.g., ticks)
	**Score 3**	**High:** Exclusively vector transmission by flying vectors (e.g., culicoides, mosquitoes)
	**Score 4**	**Very high:** more than three modes of transmission and/or airborne transmission
**D1_9**	**Environmental persistence**	
	**Score 0**	**Null:** pathogen does not survive in the environment
	**Score 1**	**Low:** only anecdotal isolation of the pathogen from the environment has been recorded
	**Score 2**	**Moderate:** The survival of the agent in the environment is limited (only temporary) and it’s dependent on certain environmental conditions such as humidity, temperature, rainfall, etc.
	**Score 3**	**High:** The survival of the agent in the environment is limited (only temporary) and NOT dependent on certain environmental conditions such as humidity, temperature, rainfall, etc.
	**Score 4**	**Very high:** agent naturally surviving in the environment (soil, water) and organic materials were it has a long term-survival
Number of drivers = 9, hence 90 points to be distributed within this domain for the intra-domain weighing		
**Domain D2. Distance to Europe**		
**D2_1**	**Current incidence (cases)/prevalence of the disease in the world**	
	**Score 0**	
	**Score 1**	Pathogen has been reported only in the countries of the Australasia (Australia, New Zealand, New Guinea and Neighbouring Pacific Islands) region
	**Score 2**	Disease was reported in countries of the Americas, Caribbean and Asia (excluding the Russian Federation)
	**Score 3**	Disease was reported/present in the African continent
	**Score 4**	Disease was reported in countries of the Mediterranean Basin, Middle East and the Russian Federation
**D2_2**	**European geographic proximity of the pathogen/disease to Europe**	
	**Score 0**	
	**Score 1**	Disease has never been present in Europe
	**Score 2**	Disease has been reported in Europe in the past but is currently exotic.
	**Score 3**	Disease is currently present in at least one European country which is NOT bordering your country
	**Score 4**	Diseases is currently present in at least one of the countries bordering your country
**D2_3**	**To your knowledge when was the disease last reported in Europe**	
	**Score 0**	More than 20 years ago
	**Score 1**	More than 10 years ago
	**Score 2**	More than 5 years ago
	**Score 3**	More than 1 year ago
	**Score 4**	Currently present in Europe
Number of drivers = 3, hence 30 points to be distributed within this domain for the intra-domain weighing		
**Domain D3. Ability to Monitor, Treat and Control the Disease**		
**D3_1**	**Ability of preventive/control measures to stop the disease from entering the country or spreading (containment of the epidemic/pandemic), excluding treatment, vaccination and vector(s)/reservoir(s) control**	
	**Score 0**	
	**Score 1**	**Very High:** Sanitary certificate; effective traceability of animals and by-products; effective disinfection measures; no contact between domestic and wild animals; effective biosecurity measures
	**Score 2**	**High:** No sanitary certificate; effective traceability of animals and by-products; effective disinfection measures; limited or incomplete possibilities to restrict contacts between domestic and wild animals; effective biosecurity measures
	**Score 3**	**Low:** No sanitary certificate; incomplete traceability of animals and by-products; ineffective disinfection measures; incomplete restriction of contacts between domestic and wild animals; ineffective biosecurity measures
	**Score 4**	**Very low:** No sanitary certificate; no traceability of animals and by-products; ineffective disinfection measures; impossibility to restrict contact between farms or between domestic and wild animals; biosecurity measures totally ineffective
**D3_2**	**Vaccine availability**	
	**Score 0**	
	**Score 1**	**Very high:** Commercialized vaccine available on a global scale (worldwide)
	**Score 2**	**High:**Local/mono-species vaccine available at a regional/national scale and/or for a targeted species (not systematically available for a global fight plan)
	**Score 3**	**Low:**Experimental vaccine, not commercialized to date; severe adverse reaction when applied; limited protector effect
	**Score 4**	**Very low:**Absence; no vaccine available on the market for a use in the species considered in the study, no experimental vaccine either
**D3_3**	**Control of reservoir(s) and/or vector(s)**	
	**Score 0**	**Null:** No vector-borne transmission and/or no reservoir(s) known to date
	**Score 1**	**Very high:** Effective. Limited reservoir(s) with limited geographical repartition, easy-to-identify; high scientific knowledge on vector(s)/reservoir(s); effective fighting measures
	**Score 2**	**High:** Limited reservoir(s)/vector(s) with limited geographical repartition; easy-to-identify, high scientific knowledge on vector(s)/reservoir(s); effective fighting measures but not applicable at a large scale; limited fighting measures
	**Score 3**	**Low:** Numerous reservoirs vectors identified with limited geographical repartition; hard to identify. Lack of scientific knowledge on vector(s)/reservoir(s). Fighting measures are poorly effective—resistances and/or negative impact on environment;
	**Score 4**	**Very low:** Numerous Vector(s)/reservoir(s)identified with wide geographic distribution; hard to identify, absence of scientific knowledge on vector(s)/reservoir(s); no effective fighting measure against vector(s) (no active molecule, resistance to measures applied)
**D3_4**	**Availability and quality of diagnostic tools in your country**	
	**Score 0**	
	**Score 1**	**Very High:** Field test(s) available and easy to use, with highly discriminating sensitivity and specificity
	**Score 2**	**High:** Tests used in local/regional laboratories by not in the field
	**Score 3**	**Low:** tests only used in *specialized* laboratories/national reference laboratory
	**Score 4**	**Very Low:** no diagnostic tools available to date
**D3_5**	**Disease is currently under surveillance overseas (OIE, EU)**	
	**Score 0**	
	**Score 1**	**Very high:** Generalized surveillance implemented by all EU Member States and worldwide surveillance (i.e. OIE reported)
	**Score 2**	**High:** Surveillance of the pathogen only EU member states
	**Score 3**	**Low:** Surveillance only in some EU member states (because they had cases of the disease) and only in some non-EU countries (not a disease reported in any international organisations)
	**Score 4**	**Very low:**Absence of surveillance of the pathogen in all EU member countries and worldwide
**D3_6**	**Eradication experience in other countries and/or your country**	
	**Score 0**	
	**Score 1**	**Very high:** Previous experience on eradication has been applied, fast and successfully
	**Score 2**	**High:** Previous experience on eradicating the disease but with some setbacks in the process
	**Score 3**	**Low:** Knowledge on eradication procedures but have never had to implement an eradication program in your country
	**Score 4**	**Very low:** It is a novel disease, first time countries are faced with a new disease to eradicate
**D3_7**	**Detection of emergence—e.g., difficulties for the farmer/veterinarian to declare the disease or clinical signs not so evident**	
	**Score 0**	
	**Score 1**	**Very high:** Disease is easily detected with clinically signs and farmers are aware of the disease and willing to notify it as soon as possible it
	**Score 2**	**High:** Disease is easily detected by the clinical signs but farmers don’t have sufficient knowledge/awareness nor interest to notify it
	**Score 3**	**Moderate:** Disease is not as easily detect by the clinical signs and farmers don’t have sufficient knowledge/awareness nor interest to notify
	**Score 4**	**Low:** The infected animal does not show any pathognomonic clinical sign(s); farmer is reluctant to declare/notify any abnormality
Number of drivers = 7, hence 70 points to be distributed within this domain for the intra-domain weighing		
**DOMAIN D4. Farm/European characteristics.**		
**D4_1**	**Mono species farms—One single farmed animal (e.g., only bovines) or multi species farms (farms with more than one species e.g., goats and bovines in the same farm/land/premises)**	
	**Score 0**	
	**Score 1**	**Negligible:** the type of farm does not influence in any form (re)emergence of the disease among the livestock population
	**Score 2**	**Low:** mono or multi species farm has a low effect on the risk of disease to emerge or re-emerge
	**Score 3**	**Moderate:** the type or types of farmed animals has a moderate effect on the emergence of the disease in your country
	**Score 4**	**High:** the type of farmed animals has a high influence for the disease to emerge and spread in your country
**D4_2**	**Farm demography/management: such as type of dairy or beef (cattle) production. For pigs—reproduction, fattening, finishing farm or both**	
	**Score 0**	
	**Score 1**	**Negligible:** population demography does not influence in any form the (re)emergence of the disease among the livestock population
	**Score 2**	**Low:** the demographic population of the farm is a low influencing factor for disease (re)emergence. e.g., Disease only clinically affects only one age strata (i.e.) new-borns, therefore adults are immune to it
	**Score 3**	**Moderate:** the demographic of the population has a moderate effect on the (re)emergence of the disease, as it can (re)emerge in more than one type of demography but other conditioning factors have to occur in conjunction
	**Score 4**	**High:** the type of demographic of the farm has a high effect on the (re)emergence of the disease as it can (re)emerge in different types of farmed animals and all types of age groups
**D4_3**	**Animal density of farms. Extensive (small holders with a few animals) *versus* intensive farming**	
	**Score 0**	
	**Score 1**	**Negligible:** animal farm density is not a risk factor for the disease to emerge in your country
	**Score 2**	**Low:** farm density (extensive or intensive) of animals has a low effect on the pathogen’s/disease (re)emergence
	**Score 3**	**Moderate:** farm density of animals in the farm (extensive v/s intensive) has a moderate effect on the emergence of pathogen/disease
	**Score 4**	**High:** farm density of animals has a high effect on the (re)emergence of pathogen/disease
**D4_4**	**Feeding practices of farms**	
	**Score 0**	
	**Score 1**	**Negligible:** Feeding practices have a negligible effect on the (re)emergence of the pathogen/disease
	**Score 2**	**Low:** Feeding practices have a low effect on the (re)emergence of the pathogen/disease
	**Score 3**	**Moderate:** Feeding practices have a moderate effect on the (re)emergence of the pathogen/disease
	**Score 4**	**High:** Feeding practices have a high effect on the (re)emergence of the pathogen/disease
**D4_5**	**Human movements among premises—Veterinarians or farm staff**	
	**Score 0**	
	**Score 1**	**Negligible:** disease is spread by other means
	**Score 2**	**Low:** movement of human staff has a low effect on the introduction or spread of the disease
	**Score 3**	**Moderate:** movement of human staff has a moderate effect on the introduction or spread of the disease
	**Score 4**	**High:** movement of human staff has a high effect on the introduction or spread of the disease
**D4_6**	**Proximity of livestock farm to wildlife and wildlife reservoirs of disease e.g., contact with wild or feral birds and animals which have been scavenging on landfill sites that contain contaminated animal products**	
	**Score 0**	
	**Score 1**	**Negligible:** Disease (re)emergence from wildlife and wildlife reservoir never reported
	**Score 2**	**Low:** Disease (re)emergence from wildlife and wildlife reservoir rarely reported
	**Score 3**	**Moderate:** Disease (re)emergence from wildlife and wildlife reservoir is documented regularly
	**Score 4**	**High:** wildlife is a reservoir for the disease and the main source of infection for livestock
**D4_7**	**Changes of land use, e.g., field fragmentation, creation of barriers, landfill sites**	
	**Score 0**	
	**Score 1**	**Negligible:** Changes in land use have a negligible effect on the (re)emergence of pathogen/disease
	**Score 2**	**Low:** changes in land use have a low effect on the (re)emergence of the disease/pathogen but need other factors (e.g., land use changes combined with higher winter temperatures)
	**Score 3**	**Moderate:** land use changes increases the availability of vectors or increases the pathogen’s survival. Also empty land can create a suitable environment for certain wildlife carrying the disease (e.g., migratory birds)
	**Score 4**	**High:** land use changes are one of the main drivers for pathogen or its vectors
Number of drivers = 7, hence 70 points to be distributed within this domain for the intra-domain weighing		
**Domain D5. Changes in Climatic Conditions**		
**D5_1**	**Influence of annual rainfall in the survival and transmission of the pathogen/disease**	
	**Score 0**	
	**Score 1**	**Negligible:** Pathogen survival and mode of transmission of the disease are not influenced by increased rainfall
	**Score 2**	**Low:** pathogen survival and mode of transmission of the disease are slightly influenced by increased rainfall
	**Score 3**	**Moderate:** pathogen survival and mode of transmission of the disease are moderately influenced by increased rainfall
	**Score 4**	**High:** pathogen survival and mode of transmission of the disease are highly influenced by increased rainfall
**D5_2**	**Influence of annual humidity in the survival and transmission of the pathogen/disease**	
	**Score 0**	
	**Score 1**	**Negligible:** Pathogen survival and mode of transmission of the disease are not influenced by increased humidity
	**Score 2**	**Low:** pathogen survival and mode of transmission of the disease are slightly influenced by increased humidity
	**Score 3**	**Moderate:** pathogen survival and mode of transmission of the disease are moderately influenced by increased humidity
	**Score 4**	**High:** pathogen survival and mode of transmission of the disease are highly influenced by increased humidity
**D5_3**	**Influence of annual temperature in the survival and transmission of the pathogen/disease**	
	**Score 0**	
	**Score 1**	**Negligible:** Pathogen survival and mode of transmission of the disease are not influenced by increased temperature
	**Score 2**	**Low:** pathogen survival and mode of transmission of the disease are slightly influenced by increased temperature
	**Score 3**	**Moderate:** pathogen survival and mode of transmission of the disease are moderately influenced by increased temperature
	**Score 4**	**High:** pathogen survival and mode of transmission of the disease are highly influenced by increased temperature
Number of drivers = 3, hence 30 points to be distributed within this domain for the intra-domain weighing		
**Domain D6. Wildlife Interface**		
**D6_1**	**Potential roles of zoo’s in the (re)emergence of the pathogen**	
	**Score 0**	
	**Score 1**	**Negligible:** The disease can be present in zoo animals, but it is not known to have been transmitted from zoo animals to livestock
	**Score 2**	**Low:** The disease can enter a zoo (e.g., with introduction of an infected exotic animal) but only accidental transmissions of the disease from zoo animals to livestock have been reported. Hence, zoos have a low effect on the (re)emergence of the disease in livestock of your country
	**Score 3**	**Moderate:** The disease can enter a zoo and be present in zoo animals but it needs a vector (biological/mechanical) for its transmission into livestock. Therefore, zoos have a moderate effect on the (re)emergence of the disease your country
	**Score 4**	**High:** Disease can be introduced to a zoo via an infected imported animal, zoo animals can carry the disease that can easily jump to livestock animals
**D6_2**	**The rural(farm)-wildlife interface**	
	**Score 0**	
	**Score 1**	**Negligible:** the disease has never (re)emerged from the narrowing of the farm-wild interface
	**Score 2**	**Low:** the disease has a low probability to (re)emerge via the livestock farm-forest interface. The disease has been known to (re)emerge from the wild bush but very rarely
	**Score 3**	**Moderate:** the disease has a moderate probability of (re)emergence via the farm/wildlife interface. Barriers ( natural or artificial) are needed to keep the disease/pathogen (re)emerging in livestock
	**Score 4**	**High:** there is a high probability for the disease to (re)emerge via the farm/forest interface. Barriers (natural or artificial) separating farms from natural forests are ineffective
**D6_3**	**Increase of autochthons (indigenous animal) wild mammals in Europe and neighbouring countries**	
	**Score 0**	**Not applicable:** disease has not been reported in wildlife
	**Score 1**	**Negligible:** the increase the autochthonous mammals population does not affect the risk of the diseases to (re)emergence
	**Score 2**	**Low:** The slight increase of autochthonous mammals can slightly increase the probably of the disease emerging
	**Score 3**	**Moderate:** The increase of wild mammals has been associated with the re-emergence of the disease
	**Score 4**	**High:** The increase of wild mammals is the only factor associated with outbreaks of the disease in livestock
**D6_4**	**Increase in endemic/migrating populations of wild birds**	
	**Score 0**	**Not applicable:** Wild/migrating birds are not a reservoir of the disease
	**Score 1**	**Negligible:** there is a negligible probability of disease (re)emerging in livestock because of an increase in populations of endemic/migrating wild birds.
	**Score 2**	**Low:** there is a low probability of the disease (re)emerging and spreading through increased populations of endemic/migrating wild birds. Disease has spread from the endemic/migrating wild birds but only accidentally or under exceptional circumstances
	**Score 3**	**Moderate:** there is a moderate probability of disease being introduced and spread through increased populations of endemic/migrating wild birds. They are hosts and in close contact with domestic livestock (i.e., poultry farms) may spread the disease
	**Score 4**	**High:** there is a high probability for a disease to (re)emerge through increased populations of wild/migrating birds. These are hosts or reservoirs of the disease
**D6_5**	**Hunting Activities: hunted animals can be brought back to where livestock is present**	
	**Score 0**	
	**Score 1**	**Negligible:** The risk of the disease/pathogen of (re)emerging in livestock due to hunting activities is practically null
	**Score 2**	**Low:** disease is present in hunted wildlife and birds and only accidental cases have been reported in livestock that have (re)emerged because of hunting. The risk of the disease/pathogen of (re)emerging in livestock due to hunting activities is practically null
	**Score 3**	**Moderate:** disease is present in hunted wildlife and birds but a certain control is established by the hunter
	**Score 4**	**High:** disease is present in hunted wildlife and birds and hunting is one of the main modes of transmission of the disease to livestock
**D6_6**	**Transboundary movements of terrestrial wildlife from other countries**	
	**Score 0**	**Not applicable:** Disease is not carried by terrestrial wildlife
	**Score 1**	**Negligible:** (re)emergence of the disease by terrestrial movements of wildlife has only been suspected but never confirmed
	**Score 2**	**Low:** There is a low probability for the disease to (re)emerge and spread through transboundary movements of terrestrial wildlife
	**Score 3**	**Moderate:** There is a moderate probability for the disease to (re)emerge and spread through transboundary movements of terrestrial wildlife
	**Score 4**	**High:** There is a high probability for the disease to (re)emerge and spread through transboundary movements of terrestrial wildlife. These are host and may spread/carry the disease along
Number of drivers = 6, hence 60 points to be distributed within this domain for the intra-domain weighing		
**Domain D7. Human Activities**		
**D7_1**	**In- and out- people movements linked to tourism**	
	**Score 0**	
	**Score 1**	**Negligible:** the movement of tourism is a negligible driver on the emergence or re-emergence of the disease
	**Score 2**	**Low:** tourism increase has a low driver of the (re)emergence of the disease
	**Score 3**	**Moderate:** tourism increase has a moderate driver for the (re)emergence of the disease. Biosecurity measures are enough to stop the entering of the pathogen
	**Score 4**	**High:** tourist movement is a high driver on the (re)emergence of a disease. Tourists are highly likely to bring the disease into your country in their belongings and biosecurity measures are insufficient to stop the pathogen
**D7_2**	**Human Immigration**	
	**Score 0**	
	**Score 1**	**Negligible:** the immigration movements are a negligible driver of the disease (re)emergence in your country
	**Score 2**	**Low:** the immigration movements are a low driver of the disease (re)emergence in your country
	**Score 3**	**Moderate:** the disease is currently present in countries where more immigrants come from and pathogen highly likely to enter through, clothes, shoes and or possession, but the current biosecurity measures in place are able to prevent the emergence of the disease in your country
	**Score 4**	**High:** the immigration movement has a high effect as a driver on the emergence or re-emergence of disease in your country. Disease is highly likely to emerge using this route as biosecurity measures are not enough to avoid emergence of the disease
**D7_3**	**Transport movements: more specifically commercial flights, commercial transport by ships, cars or military (excluding transport vehicles of live animals)**	
	**Score 0**	
	**Score 1**	**Negligible:** the role of commercial movements as a driver on the (re)emergence of the disease in your country is negligible
	**Score 2**	**Low:** the role of commercial movements as a driver on the (re)emergence of the disease in your country is low. It is easily preventable by implementing biosecurity measures
	**Score 3**	**Moderate:** the role of commercial movements as a driver on the (re)emergence of a disease in your country is moderate. Disease can be prevented if biosecurity measures are tightened
	**Score 4**	**High:** the role of commercial movements as a driver on the (re)emergence of a disease in your country is high. Disease is hard to control via the current biosecurity measures
**D7_4**	**Transport vehicles of live animals**	
	**Score 0**	
	**Score 1**	**Negligible:** the role of transport vehicles of live animals as a driver for the (re)emergence of the disease in your country is negligible
	**Score 2**	**Low:** the role of transport vehicles of live animals as a driver for the (re)emergence of the disease in your country is low
	**Score 3**	**Moderate:** the role of transport vehicles of live animals as a driver for (re)emergence of the disease in your country is moderate
	**Score 4**	**High:** the role of transport vehicles of live animals as a driver for (re)emergence of the disease in your country is high
**D7_5**	**Bioterrorism potential**	
	**Score 0**	
	**Score 1**	**Negligible:** the role of bioterrorism as a driver for a disease to (re)emerge is negligible: agent is available but difficult to handle or has a low potential of spread or generates few economic consequences
	**Score 2**	**Low:** the role of bioterrorism as a driver for a disease to (re)emerge is low: agent is available and easy to handle by professionals and labs but has a low spread
	**Score 3**	**Moderate:** the role of bioterrorism as a driver for a disease to (re)emerge is moderate: agent available and easy to handle by professionals and labs and rapidly spreads
	**Score 4**	**High:** the role of bioterrorism as a driver for a disease to (re)emerge is high: Agent is available and easy to handle by individuals and rapidly spreads
**D7_6**	**Inadvertent release of an exotic infectious agent from a containment facility e.g., Laboratory**	
	**Score 0**	
	**Score 1**	**Negligible:** the pathogen is not currently present in any laboratory
	**Score 2**	**Low:** the pathogen is present in a containment facility but its release is very unlikely as it is very easily contained
	**Score 3**	**Moderate:** the pathogen is present in a containment facility and its release can occur as not easily contained
	**Score 4**	**High:** pathogen is handled in a risk 3 or 4 laboratory (BSL3 or BSL4) in the country. It can leave the facility if the correct biosecurity measures are not implemented correctly and easily spread to livestock
Number of drivers = 6, hence 60 points to be distributed within this domain for the intra-domain weighing		
**Domain D8. Economic and Trade Activities**		
**D8_1**	**Decrease of resources allocated to the disease surveillance**	
	**Score 0**	
	**Score 1**	**Negligible:** resources allocated to the disease surveillance have no effect on the (re)emergence of the disease in your country Disease has never been under surveillance
	**Score 2**	**Low:** resources allocated to the disease surveillance have a low effect on the (re)emergence of the disease in your country Disease has been under surveillance in the past and no change has happened after surveillance has been stopped
	**Score 3**	**Medium:** resources allocated to the disease surveillance have a moderate effect on the (re)emergence of the disease in your country Disease is under passive surveillance (reported only when observed) but with no need to further increase its surveillance
	**Score 4**	**High:** resources allocated to the disease surveillance have a high effect on the (re)emergence of the disease in your country Disease needs to be under active and passive surveillance as its (re)emergence can easily occur, therefore if its surveillance decreases it’s highly likely to (re)emerge
**D8_2**	**Modification of the disease status (i.e., reportable disease becoming not reportable) or change in screening frequency due to a reduced national budget**	
	**Score 0**	
	**Score 1**	**Negligible:** modification of the disease status due to a reduced national budget has a negligible effect on the (re) emergence of the disease in your country
	**Score 2**	**Low:** modification of the disease status due to a reduced national budget has a low effect on the (re) emergence of the disease in your country
	**Score 3**	**Moderate:** modification of the disease status due to a reduced national budget has a moderate effect on the (re) emergence of the disease in your country
	**Score 4**	**High:** modification of the disease status due to a reduced national budget has a high effect on the (re) emergence of the disease in your country
**D8_3**	**Decrease of resources allocated to the implementation of biosecurity measures at border controls (e.g., harbors or airports)**	
	**Score 0**	
	**Score 1**	**Negligible:** decreasing the resources allocated to the implementation of biosecurity measures has a negligible effect on the (re)emergence of the disease in your country. Disease has never been detected in the past in a harbor or airport
	**Score 2**	**Low:** decreasing the resources allocated to the implementation of biosecurity measures has a low effect on the (re)emergence of the disease in your country. The disease has been suspected to have entered other countries because of deficient biosecurity at border controls
	**Score 3**	**Medium:** decreasing the resources allocated to the implementation of biosecurity measures has a moderate effect on the (re)emergence of the disease in your country. The disease has been introduced in other countries because of deficient biosecurity at border controls
	**Score 4**	**High:** decreasing the resources allocated to the implementation of biosecurity measures highly increases the risk of (re)emergence of the disease in your country. In the past, the disease has been introduced in other countries and in your country because of deficient biosecurity at border controls
**D8_4**	**Most likely influence of (il)legal movements of live animals (livestock, pets, horses etc) from neighbouring/European Union member states (MS) for the disease to (re)emerge in your country**	
	**Score 0**	
	**Score 1**	**Negligible:** (il)legal movements of live animals (livestock, pets, horses etc) from neighbouring/European Union MS have a negligible influence on the pathogen/disease (re)emergence in your country
	**Score 2**	**Low:** (il)legal movements (livestock, pets, horses etc) from neighbouring/European Union MS have a low influence on the pathogen/disease (re)emergence in your country
	**Score 3**	**Moderate:** (il)legal movements (livestock, pets, horses etc) from neighbouring/European Union MS have a moderate influence on the pathogen/disease (re)emergence in your country
	**Score 4**	**High:** (il)legal movements (livestock, pets, horses etc.) from neighbouring/European Union MS have a high influence on the pathogen/disease (re)emergence in your country
**D8_5**	**Most likely influence of (il)legal movements of pets from Third countries for the disease to (re)emerge in Europe**	
	**Score 0**	
	**Score 1**	**Negligible:** increased (il)legal imports of animal subproducts such as skin, meat and edible products from EU member states have a negligible influence on the pathogen/disease (re)emergence in your country
	**Score 2**	**Low:** increased (il)legal imports of animal subproducts such as skin, meat and edible products from EU member states have a low influence on the pathogen/disease (re)emergence in your country
	**Score 3**	**Moderate:** increased (il)legal imports of animal subproducts such as skin, meat and edible products from EU member states have a moderate influence on the pathogen/disease (re)emergence in your country
	**Score 4**	**High:** increased (il)legal imports of animal subproducts such as skin, meat and edible products from EU member states have a high influence on the pathogen/disease (re)emergence in your country
**D8_6**	**Most likely influence of increased (il)legal imports of non-animal products such as tires, wood, furniture from EU member states for the disease/pathogen to (re)emerge in your country**	
	**Score 0**	
	**Score 1**	**Negligible:** (il)legal movements of live animals (livestock, pets, horses etc) from Third countries have a negligible influence on the pathogen/disease (re)emergence in your country
	**Score 2**	**Low:** (il)legal movements of live animals (livestock, pets, horses etc) from Third countries have a low influence on the pathogen/disease (re)emergence in your country
	**Score 3**	**Moderate:** (il)legal movements of live animals (livestock, pets, horses etc) from Third countries have a moderate influence on the pathogen/disease (re)emergence in your country
	**Score 4**	**High:** (il)legal movements of live animals (livestock, pets, horses etc) from Third countries have a high influence on the pathogen/disease (re)emergence in your country.
**D8_7**	**Most likely influence of (il)legal movements of live animals (livestock, pets, horses etc) from Third countries for the disease to (re)emerge in your country**	
	**Score 0**	
	**Score 1**	**Negligible:** (il)legal movements of live animals (livestock, pets, horses etc) from Third countries have a negligible influence on the pathogen/disease (re)emergence in your country
	**Score 2**	**Low:** (il)legal movements of live animals (livestock, pets, horses etc) from Third countries have a low influence on the pathogen/disease (re)emergence in your country
	**Score 3**	**Moderate:** (il)legal movements of live animals (livestock, pets, horses etc) from Third countries have a moderate influence on the pathogen/disease (re)emergence in your country
	**Score 4**	**High:** (il)legal movements of live animals (livestock, pets, horses etc) from Third countries have a high influence on the pathogen/disease (re)emergence in your country
**D8_8**	**Most likely influence of increased imports of animal sub-products such as skin, meat and edible products from Third countries, for the disease to (re)emerge in your country**	
	**Score 0**	
	**Score 1**	**Negligible:** Increased imports of animal subproducts such as skin, meat and edible products from Third countries have a negligible influence on the pathogen/disease (re)emergence in your country
	**Score 2**	**Low:** Increased imports of animal subproducts such as skin, meat and edible products from Third countries have a low influence on the pathogen/disease (re)emergence in your country
	**Score 3**	**Moderate:** Increased imports of animal subproducts such as skin, meat and edible products from Third countries have a moderate influence on the pathogen/disease (re)emergence in your country
	**Score 4**	**High:** Increased imports of animal subproducts such as skin, meat and edible products from Third countries have a high influence on the pathogen/disease (re)emergence in your country
**D8_9**	**Most likely influence of increased (il)legal imports of non-animal products such as tires, wood, furniture from Third countries, for the disease to (re)emerge in your country**	
	**Score 0**	
	**Score 1**	**Negligible:** increased (il)legal imports of non-animal products such as tires, wood, furniture from Third countries have a negligible influence on the pathogen/disease (re)emergence in your country
	**Score 2**	**Low:** increased (il)legal imports of non-animal products such as tires, wood, furniture from Third countries have a low influence on the pathogen/disease (re)emergence in your country
	**Score 3**	**Moderate:** increased (il)legal imports of non-animal products such as tires, wood, furniture from Third countries have a moderate influence on the pathogen/disease (re)emergence in your country
	**Score 4**	**High:** increased (il)legal imports of non-animal products such as tires, wood, furniture from Third countries have a high influence on the pathogen/disease (re)emergence in your country
Number of drivers = 9, hence 90 points to be distributed within this domain for the intra-domain weighing		

## Appendix C

Guidance letter for the expert elicitation.

      **Ranking criteria of emerging bovine besnoitiosis in Belgium**

                    **(Experts’ opinion)**

Dear Colleague

This special request is related to the emergence of bovine besnoitiosis in Belgium.

The objective of the present study is to understand more the drivers of emergence of the bovine besnoitiosis in Belgium. The questionnaire was prepared in order to present different criteria of interest and summarized in a total of 50 drivers. For each driver, scores are given with a corresponding definition. Drivers are grouped by category (N = 8), each category in one spreadsheet. After the scoring the weight of each driver for a specific category of drivers and for each category are performed.

**Objective of the questionnaire**

We would like your expert opinion on the drivers of emerging bovine besnoitiosis in Europe.

Hence, to answer on the basis of “how likely is it for bovine besnoitiosis in Belgium in response to the different drivers”.

**How to fill the questionnaire**

In the attached Excel Questionnaire, there are **9** Spreadsheets. The first **8** correspond to the 8 categories of drivers and the **9th** to the Intra-category weighing.

1. Disease/pathogen characteristics: 9 criteria
2. Distance to Europe (spatial-temporal scales): 3 criteria
3. Ability to monitor, treat and control the disease: 7 criteria
4. Farm/European characteristic: 7 criteria
5. Climatic conditions: 3 criteria
6. Wildlife interface: 6 criteria
7. Human activities: 6 criteria
8. Economy and trade activities: 9 criteria
9. Intra-category weighing

**Actions to be done:**

1) Score and balance each driver within each category of drivers:

■ Please give a score according to what you estimate is the importance of each driver in the (re)-emergence of specific disease(s).
■ After the scoring, please balance each driver for each category of drivers. Balancing the criteria will rely on the distribution of points between the different proposed criteria under each category. The total number of points to be distributed among the drivers is specified for each category (each spreadsheet). e.g., category pathogen characteristics total 90 points; distance of outbreaks (spatial-temporal scales) a total of 30 points to be distributed.

2) Intra-category weighing: The last step of the process will consist in the distribution of **80** points between the **8 categories** of criteria (Pathogen characteristics, distance of outbreaks, etc.). This is on the 9th spreadsheet. The distribution will depend on which is believed to be the strongest category of drivers.

As an expert in the field, your collaboration will help us a lot for the good course of the project.

Thank you in advance for your collaboration and for the time spent in filling the file **before the Date.**

For any question, not hesitate to contact claude.saegerman@uliege.be

Kind Regards,

         **Professor Claude Saegerman**

       Research Unit in Epidemiology and Risk

       Analysis applied to Veterinary Sciences

    Fundamental and Applied Research for Animal & Health

      Department of Infectious and Parasitic Diseases

         Faculty of Veterinary Medicine

           University of Liège

            Quartier Vallée 2

          Avenue de Cureghem 7A

           4000 Liège Sart-Tilman

             **BELGIUM**

          Tél.: + 32-(0)4-366-45-79

        E-mail: claude.saegerman@uliege.be
